# Preclinical studies using cisplatin/carboplatin to restore the Enzalutamide sensitivity via degrading the androgen receptor splicing variant 7 (ARv7) to further suppress Enzalutamide resistant prostate cancer

**DOI:** 10.1038/s41419-020-02970-4

**Published:** 2020-11-02

**Authors:** Fu-Ju Chou, ChangYi Lin, Hao Tian, WanYing Lin, Bosen You, Jieyang Lu, Deepak Sahasrabudhe, Chi-Ping Huang, Vanessa Yang, Shuyuan Yeh, Yuanjie Niu, Chawnshang Chang

**Affiliations:** 1 George Whipple Lab for Cancer Research, Departments of Pathology, Urology, Radiation Oncology and The Wilmot Cancer Institute, University of Rochester Medical Center, Rochester, NY 14642 USA; 2 Tianjin Institute of Urology, Tianjin Medical University, Tianjin, 300211 China; 3 Department of Urology, The 4th Affiliated Hospital of Harbin Medical University, Harbin, 150001 China; 4 Sex Hormone Research Center and Department of Urology, China Medical University and Hospital, Taichung, 404 Taiwan

**Keywords:** Cancer therapeutic resistance, Biologics

## Abstract

The FDA-approved anti-androgen Enzalutamide (Enz) has been used successfully as the last line therapy to extend castration-resistant prostate cancer (CRPC) patients’ survival by an extra 4.8 months. However, CRPC patients eventually develop Enz-resistance that may involve the induction of the androgen receptor (AR) splicing variant ARv7. Here we found that Cisplatin (Cis) or Carboplatin, currently used in chemotherapy/radiation therapy to suppress tumor progression, could restore the Enz sensitivity in multiple Enz-resistant (EnzR) CRPC cells via directly degrading/suppressing the ARv7. Combining Cis or Carboplatin with Enz therapy can also delay the development of Enz-resistance in CRPC C4-2 cells. Mechanism dissection found that Cis or Carboplatin might decrease the ARv7 expression via multiple mechanisms including targeting the lncRNA-Malat1/SF2 RNA splicing complex and increasing ARv7 degradation via altering ubiquitination. Preclinical studies using in vivo mouse model with implanted EnzR1-C4-2 cells also demonstrated that Cis plus Enz therapy resulted in better suppression of EnzR CRPC progression than Enz treatment alone. These results not only unveil the previously unrecognized Cis mechanism to degrade ARv7 via targeting the Malat1/SF2 complex and ubiquitination signals, it may also provide a novel and ready therapy to further suppress the EnzR CRPC progression in the near future.

## Introduction

Prostate cancer (PCa) is the second leading cause of death among men in the United States^1^. It is estimated that there will be 191,930 new cases of PCa and 33,330 associated deaths worldwide in 2020^2^. Androgen deprivation therapy (ADT), the current standard treatment for advanced PCa via reducing androgen synthesis or preventing androgens from binding to the androgen receptor (AR), has little effect to reduce AR expression^3–6^.

Enzalutamide (Enz) could suppress the castration resistant PCa (CRPC) and might extend patients overall survival by 4.8 months^7^. However, patients still eventually develop Enz-resistance^8^. Clinical studies indicated the failure of ADT with Enz (ADT-Enz) treatment might be linked to the AR splicing variant ARv7^8,9^, a process that involves the splicing of full-length AR pre-mRNA^9,10^ and altering the RNA splicing pattern^11^. While other mechanistic studies also indicated that the development of Enz-resistance could also be due to Glucocorticoid receptor (GR) activation, AR gain, ligand binding domain mutations, or alternative AR variants^12–14^, the emergence of the ARv7 splice variant remains the most interesting explanation, supported by clear and strong evidence from human clinical sample surveys^15^.

Cisplatin (Cis) is the first FDA-approved platinum compound for cancer treatment^16^ and is widely used as a chemotherapeutic reagent to suppress many solid tumors^17^. Early mechanistic studies aimed at understanding how Cis might suppress tumor progression focused on its ability to alter the DNA damage-repair (DDR) cellular functions^17^, yet its linkage to alter AR function remains unclear.

Here we found Enz combined with Cis could to restore/increase Enz sensitivity via targeting the Malat1/SF2 RNA splicing signals to suppress the generation of the ARv7 mRNA or via altering the ubiquitination of ARv7 protein. These unexpected findings for the new Cis mechanism may help us to quickly develop a novel and ready therapy to suppress progression due to Enz-resistance to further extend CRPC patients’ survivals.

## Materials and methods

### Generation of acquired Enz resistant (EnzR) CRPC cell models

C4-2 and CWR22Rv1, were obtained from the American Type Culture Collection (ATCC) and maintained in RPMI 1640 media (#90-022-PB, CORNING, Corning, NY, USA) supplemented with 10% fetal bovine serum. EnzR1_C4-2 clone was selected by culturing cells with Enz in a dose-escalation manner with the initial culture at 10 μM Enz, and then gradually increased to 20 μM Enz. Cells proliferation rates were analyzed by MTT assays monthly. The process of acquiring drug resistance took around 12 months. EnzS4_C4-2B and EnzR4_C4-2B were obtained from from Dr. Allen Gao’s lab.

### MTT cell proliferation assay

Cells were seeded in 24-well plates (5 × 10^3^ cells per well) and cultured for 0, 2, 4, and 6 days. Cells were harvested, and absorbance (at 570 nM O.D.) were calculated and recorded after incubating with yellow tetrazolium MTT (3-(4, 5-dimethylthiazolyl-2)-2,5-diphenyltetrazolium bromide agent at 37 °C for 30 min and dissolving in DMSO.

### AR degradation and ubiquitination

For protein degradation, EnzR_C4-2 cells were treated with 1 μg/ml Cisplatin for 6 h. MG132 was added for another 4 h. For ubiquitination analysis, HEK293T cells were grown in DMEM media, and pGFP-ubiquitin or pAR were transiently transfected into cells. After 48 h of transfection, cells were treated with 1 μg/ml Cis for 6 h. MG132, was added for another 4 h. Cell extracts were analyzed for AR degradation or AR-ubiquitination using western blot.

### Circulating tumor cells (CTC) collection and isolation

5–10 ml of PCa patients’ blood were collected in EDTA tubes. The isolation steps followed the AdnaTest ProstateCancerSelect (Cat No. 395032 Qiagen, Hilden, Germany), and AdnaTest ProstateCancerDetect (Cat No. 396032, Qiagen) instructions. The total mRNA was amplified by cDNA synthesis kit (MessageBooster, Cat No. MB060124, Lucigen, Middleton, WI, USA). The samples were obtained after patients signed Informed Consent Agreements.

### RNA extraction and quantitative real-time PCR analysis

Total RNAs were isolated using Trizol reagent (Invitrogen, Grand Island, NY), or adna kit (for CTC samples) and 2 µg of total RNA was subjected to reverse transcription using Superscript III transcriptase (Invitrogen). Quantitative real-time PCR (qRT-PCR) was conducted using a Bio-Rad CFX96 system with SYBR green to determine the mRNA expression level of a gene of interest. Expression levels were normalized to the expression of *GAPDH* or *RPL13A* (for CTCs samples).

### Western blot

Cells were lysed in lysis buffer and proteins (50 µg) were separated on 10% SDS/PAGE gel and then transferred onto PVDF membranes (Millipore, Billerica, MA). After blocking membranes with 5% non-fat milk, they were incubated with appropriate dilutions of specific primary antibodies, anti-AR (N-20, sc-816, SCBT, Dallas, TX, USA), anti-GFP (sc-9996, SCBT), anti-GAPDH (sc-47724, SCBT), anti-α-tubulin (sc-8035, SCBT), the blots were incubated with HRP-conjugated secondary antibodies and visualized using ECL system (Thermo Fisher Scientific, Rochester, NY).

### Xenograft mice model

1 × 10^6^ parental EnzR3_22Rv-1 cells were subcutaneously injected with matrigel (1:1) into the right hip of nude mice. After tumors grew to ~400 mm^3^, the mice were randomly separated to 4 groups, and then treated with DMSO, Cis (3.5 mg/kg, 2 times a week), Enz (30 mg/kg, every other day) or Cis plus Enz for 20 days. Tumor sizes were measured with calipers every 5 days, and the volume of tumor calculated as follows: [(short axis^2^ × long axis)/2]. Based on preliminary data (3 mice data), if *p* < 0.05 and power = 90%, each group needs at least 4 mice. A sample will be considered to be excluded when it is 1.5 times greater than the quartile interval. Day 20 results were analyzed by One-way ANOVA. The entire Animal procedure followed UCAR regulations and was accredited by the Association for Assessment and Accreditation of Laboratory Animal Care International (AAALAC).

### Statistics

All experiments were performed at least 3 times with data points in triplicate. All statistical analyses were carried out with GraphPad Prism (GraphPad Software, San Diego, CA). The data values were presented as the mean ± S.D. (except QPCR is presented as mean ± SEM). Differences in mean values between two groups were analyzed by two-tailed unpaired Student’s *t* test. Multiple comparison was analyzed by One-way ANOVA, *p* ≤ 0.05 was considered statistically significant.

## Results

### Cisplatin or Carboplatin degrades the full-length AR (fAR) and AR variants in EnzR CRPC cells

After PCa patients developed CRPC, the current standard therapy includes either docetaxel (Doc) chemotherapy or ADT-Enz to prevent androgens from binding to AR (or with ABI to prevent androgen biosynthesis)^18^. However, most patients may still develop Enz-resistance (after an average of 4.8 months treatment)^7,18^, and recent studies indicated that the development of Enz-resistance might involve the induction of the AR splicing mutant/variant, ARv7, which could still transactivate AR at the castration level of androgens^8,19^. Knowing how to target this Enz-induced ARv7 may help in the development of a novel therapy to overcome the Enz-resistance to further improve CRPC patients’ survival rates.

The Cis (and its derivative Carboplatin with fewer side effects) is currently used widely to suppress various tumors due to its capacity to cross-link the DNA to trigger apoptosis and/or alter the DNA damage-repair (DDR) signals^20,21^. The linkage of Cis or Carboplatin to alterations of the ARv7 expression, however, remains unclear.

**Fig. 1:**
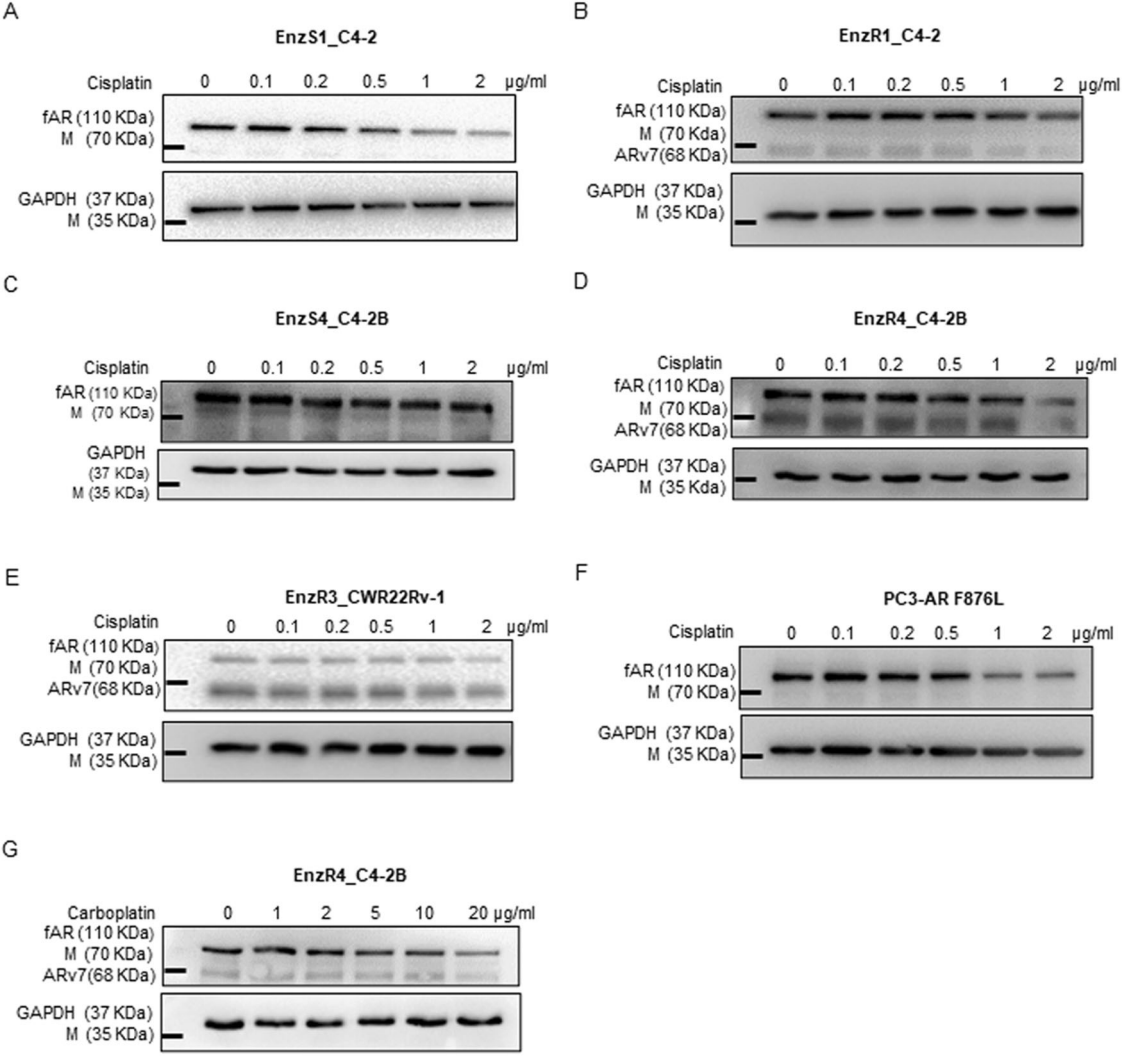
Cis/Carboplatin degrades AR wild type, mutant, and ARv7 expression and cleaved PARP in CRPCs. We established 1 EnzR PCa cell line in C4-2 cells (named as EnzR1_C4-2) and obtained EnzR4_C4-2B from another research group. **a–d** The a EnzS1_C4-2, **b** EnzR1_C4-2, **c** EnzS4_C4-2B, and **d** EnzR4_C4-2B cells were treated with increasing dosages of Cis for 24 h for fAR/ARv7 expression using Western blot (WB). **e** EnzR3_CWR22Rv1 and **f** PC3-AR-F876L cells were treated with increasing dosages of Cis for 24 h for fAR/ARv7 expression using WB. **g** EnzR4_C4-2B cells were treated with Carboplatin for 24 h for protein expression levels of fAR and ARv7 using WB.

We first established the EnzR CRPC cell line from C4-2 cells (named EnzR1_C4-2, with the original parental C4-2 cells named EnzS1_C4-2) (Fig. 1a). We then added different doses of Cis to this EnzR1_C4-2 cell line and found Cis could degrade ARv7 (and full-length AR, fAR) at the protein level (Fig. 1b). Similar results were also obtained when we replaced EnzS1_C4-2/EnzR1_C4-2 cells with other pairs of EnzS/EnzR cells, EnzS4_C4-2/EnzR4_C4-2B cells (Fig. 1c, d), or EnzR3_CWR22Rv1 (CWR22Rv1 cells are naturally EnzR) cells (Fig. 1e).

Interestingly, we found that Cis could also decrease the expression/abundance of the AR mutant AR F876L that was also induced by the Enz treatment^17^ in the mutated PC3-AR F876L cells (Fig. 1f).

**Fig. 2:**
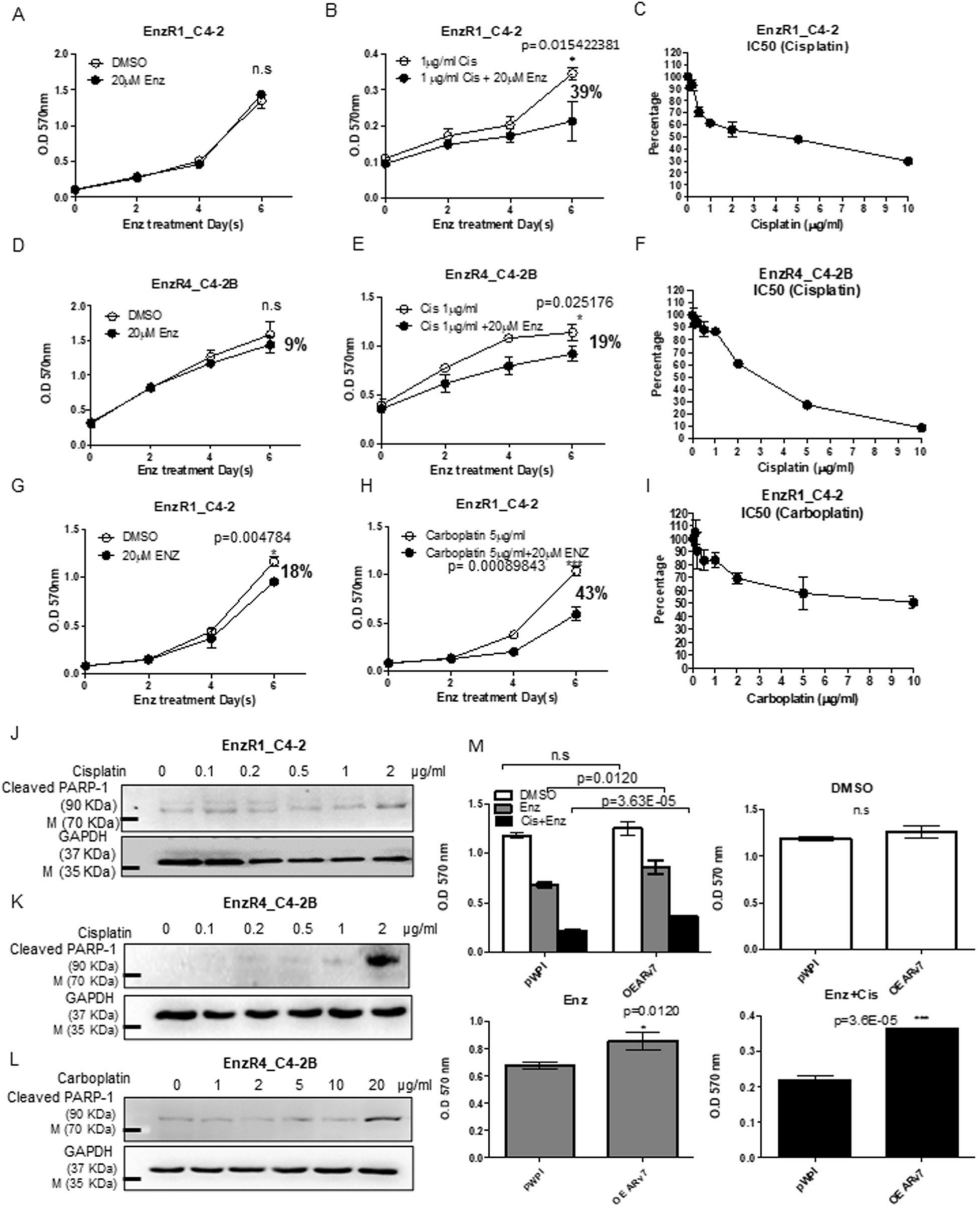
Cisplatin restores Enzalutamide sensitivity in the EnzR CRPC cells. **a–c** Cell survival rates of EnzR1_C4-2 cells treated with **a** DMSO or 20 μM Enz, **b** 1 μg/ml Cis or Cis (1 μg/ml) + Enz (20 μM), and **c** IC50 of Cis. **d–f** Survival rates of EnzR4_C4-2B treated with **d** DMSO or Enz (20 μM), **e** Cis (1 μg/ml) or Cis (1 μg/ml) + Enz (20 μM), and **f** IC50 of Cis, **g**–**i** Survival rate of EnzR1_C4-2 cells under **g** DMSO or Enz (20 μM), **h** Carboplatin (5 μg/ml) or Carboplatin + Enz (20 μM) and **i** IC50 of Cis. **j–l** Protein levels of cleaved PARP in **j** EnzR1_C4-2 cells and in **k** EnzR4_C4-2B cells that were treated with Cis for 24 h, and in **l** EnzR4_C4-2B cells that were treated with Carboplatin for 24 h. **m** Cell survival rates in EnzR1_C4-2 cells transfected with pWPI vector or OEARv7 and treated with/without Enz 10 μM + 1 μg/ml Cis (upper left), DMSO (upper right), 10 μM Enz (lower left) and Enz 10 μM + 1 μg/ml Cis (lower right). All the MTT results were performed in triplicate. Data presented as Mean ± S.D. *P < 0.05, ***P < 0.001 or n.s=not significant.

Importantly, when replacing Cis with another lower nephrotoxicity platinum-based chemotherapeutic agent, Carboplatin, we found Carboplatin could still degrade ARv7 (and fAR) (Fig. 1g), before induction of apoptosis (see Fig. 2l).

Together, the results shown in Fig. 1a–g suggest that Cis and Carboplatin may degrade ARv7 and AR mutants of AR-F876L at low doses that have minimal effects on the induction of apoptosis in multiple EnzR CRPC cells.

### Cisplatin-degraded ARv7 restores the Enz sensitivity in EnzR CRPC cells

We then applied the MTT proliferation assay to examine the consequences of Cis-degraded ARv7 on altering the Enz-sensitivity, and results revealed that treating with 20 μM Enz resulted in little suppression of the EnzR1_C4-2 growth (Fig. 2a). In contrast, treating with a low dose of Cis (see IC50 in Fig. 2c) with 20 μM Enz led to suppress EnzR1_C4-2 growth (Fig. 2b). Similar results were also obtained when we replaced EnzR1_C4-2 with EnzR4_C4-2B cells (Fig. 2d–f). We also found similar results when we replaced the Cis with carboplatin showing low dose of carboplatin plus Enz could suppress EnzR1_C4-2 growth (Fig. 2g–i).

Importantly, we found those low doses of Cis or Carboplatin had little effect on activating the cleaved PARP-1, the marker of the apoptotic pathway (Fig. 2j–l), suggesting Cis and Carboplatin effects to suppress the EnzR cell growth are not through altering cross-linking of DNA to trigger cell apoptosis.

Finally, results from the interruption assay also revealed that overexpressing (OE) ARv7 (OEARv7) back into the EnzR1_C4-2 cells also led these cells to become more resistant to Cis treatment compared with the control group (Fig. 2m, upper right). As expected, compared to DMSO control (Fig. 2m, upper right) overexpressing the EnzR1_C4-2 cells with ARv7 can partially reverse the Enz and Cis+Enz effect (Fig. 2m, lower left and right).

Together, the results from Fig. 2a–m suggest that Cis-mediated degradation of ARv7 can restore the Enz-sensitivity to further suppress the EnzR CRPC cell proliferation.

### Cisplatin delays the development of Enz-resistance in CRPC cells treated with Enz

**Fig. 3:**
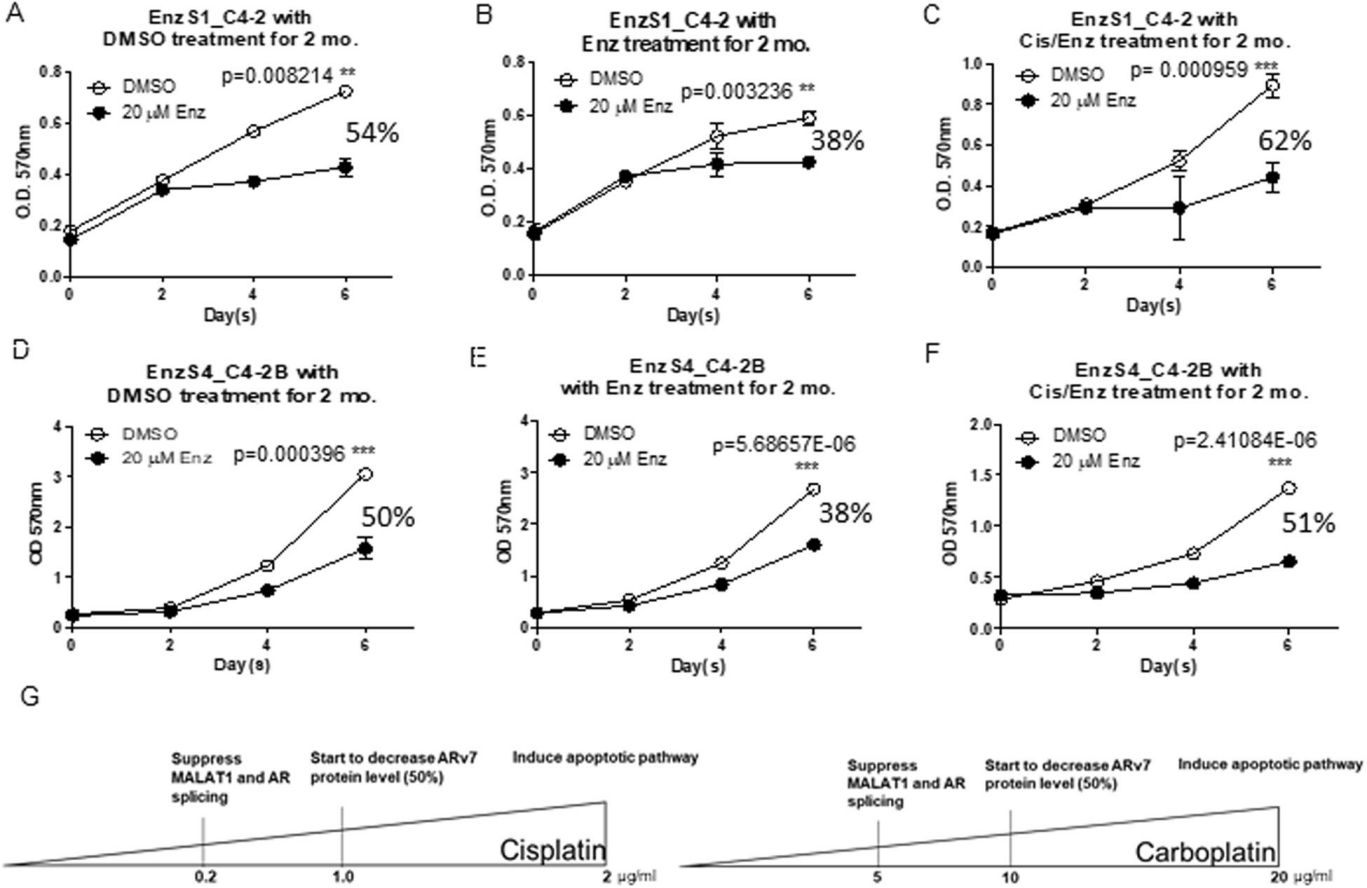
Cisplatin postpones/delays Enzalutamide-resistance development. **a**–**c** Cell survival rates of EnzS1_C4-2 cells treated with **a** DMSO, **b** 10 μM Enz or **c** 10 μM Enz + 0.2 μg/ml Cis for 2 months, then challenged with 20 μM Enz for 6 days for MTT assays. **d**–**f** Cell survival rates of EnzS4_C4-2B cells treated with **d** DMSO, **e** 10 μM Enz or **f** 10 μM Enz + 0.2 μg/ml Cis for 2 months, then challenged with 20 μM Enz for 6 days for MTT assays. Enz sensitivity was analyzed by measuring proliferation rates using MTT assays. **g** Schematic of PCa cells responses under different concentration of Cis and Carbopltin treatment. All the MTT results were performed in triplicate. Data presented as Mean ± S.D. *P < 0.05, **P < 0.01, ***P < 0.001.

In addition to restoring the Enz sensitivity to further suppress the EnzR cell proliferation, we were interested to see if Cis-degraded ARv7 can also delay the development of Enz-resistance in the CRPC cells treated with Enz. We first treated parental EnzS1_C4-2 cells with 5-10 μM Enz with a low dose of Cis for 1-2 months and then challenged the cells with 20 μM Enz for 2, 4, and 6 days followed by MTT assays. The results revealed that adding Enz alone in EnzS1_C4-2 cells for 2 months led to decrease the Enz sensitivity from 54 to 38% (Fig. 3a, b), yet 10 μM Enz + 0.2 μg/ml Cis led to delay the development of Enz-resistance (from 54 to 62% as compared to 54 to 38%) (Fig. 3c). Similar results were also obtained (from 50 to 51% as compared to 50 to 38%) when we replaced EnzS1_C4-2 cells with EnzS4_C4-2B cells (Fig. 3d–f).

Together, the results shown in Fig. 3a–f suggest that Cis can also delay the development of Enz-resistance in the CRPC cells treated with Enz via suppressing ARv7 expression. The PCa cells have similar responses to Cis and Carboplatin treatment, with 0.2 μg/ml of Cis or 5 μg/ml Carboplatin suppressing Malat1 expression, 1 μg/ml of Cis or 10 μg/ml Carboplatin decreasing AR/ARv7 protein expression by ~50%, and 2 μg/ml of Cis or 20 μg/ml Carboplatin could trigger the apoptotic pathway **(**Fig. 3g**)**.

### Mechanistic dissection of Cis-mediated decrease of ARv7 protein expression: via altering the ARv7 protein stability

**Fig. 4:**
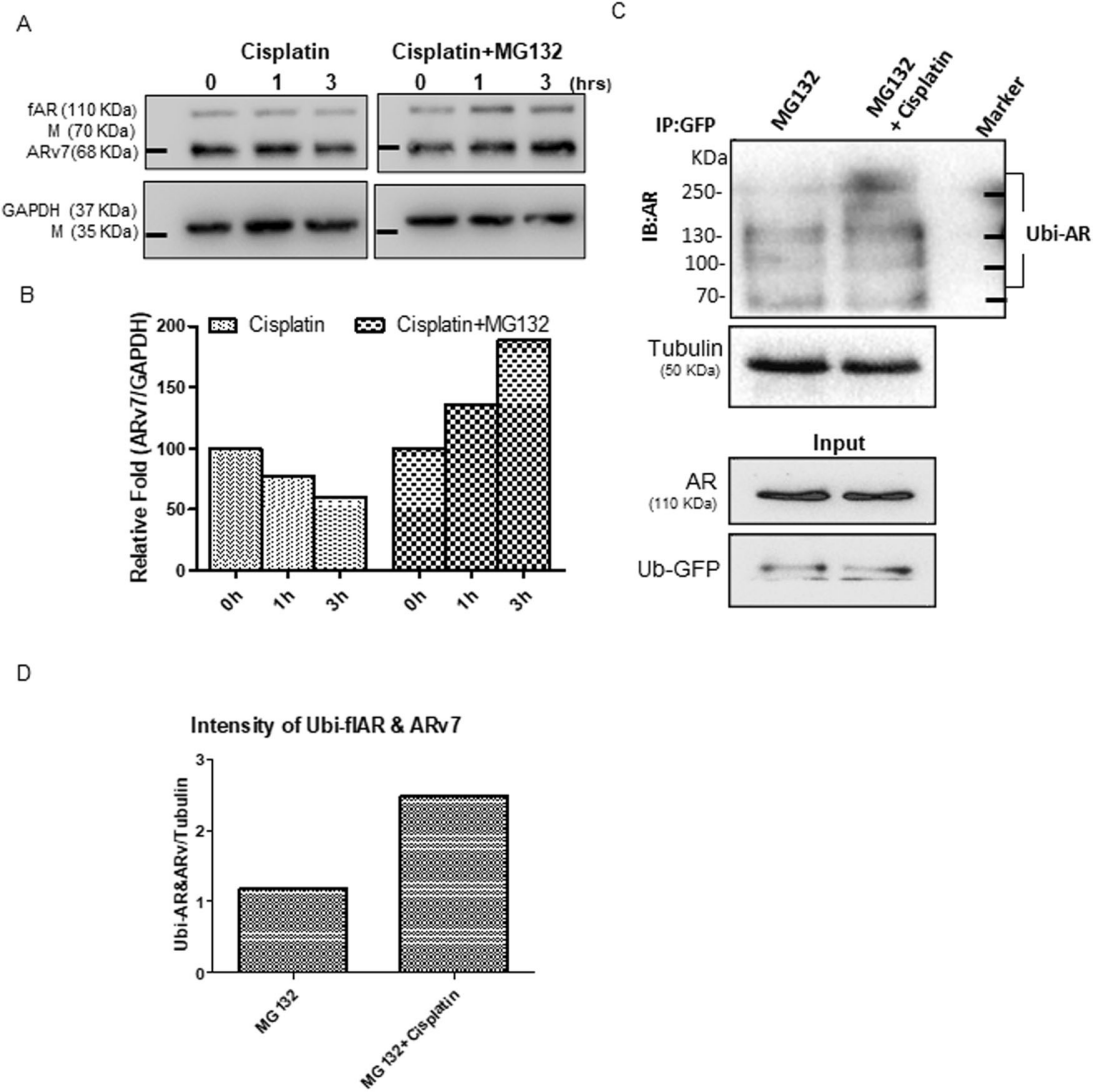
Cisplatin degrades ARv7 expression through increasing AR ubiquitination in EnzR C4-2 cells. **a** Western blot and **b** quantification of ARv7 in EnzR1_C4-2 cells which were treated with 1 μg/ml cisplatin and 1 μg/ml cisplatin for 6 h plus MG132 for another 6 h. **c** Ubiquitination levels of GFP /AR in 293 T cells transfected with Ubi-GFP and AR, which were treated with/without 1 μg/ml Cisplatin for 6 h and treated with MG132 for another 4 h. Protein was extracted and analyzed using Western blot. **d** Quantification of Ubi-flAR/ARv7.

To dissect the mechanisms why Cis can decrease ARv7 (and AR-F876L) protein expression, we first assayed the Cis effects on ARv7 (and AR876) protein stability by treating with proteasome inhibitor MG132, in EnzR3_CWR22Rv1 cells. The results revealed that Cis could degrade ARv7 after 3 hr treatment (Fig. 4a left), and treating with MG132 attenuated or inhibited the effect of Cis on ARv7 degradation (Fig. 4a right, quantification in Fig. 4b). We then examined whether ubiquitination is involved in AR/ARv7 degradation. We first added AR and ubiquitin-GFP in 293 T cells and treated with MG132, and then applied the immunoprecipitation assay to examine the Cis effect on AR-ubiquitination. The results revealed that Cis could increase the AR-ubiquitin complex (Fig. 4c, quantification in Fig. 4d).

Together, the results in Fig. 4a–d suggest that Cis can degrade ARv7 via altering the protein stability through increasing ubiquitination of AR.

### Cis can decrease ARv7 protein expression by altering the ARv7 mRNA expression through modulating the Malat1/SF2 RNA splicing complex

**Fig. 5:**
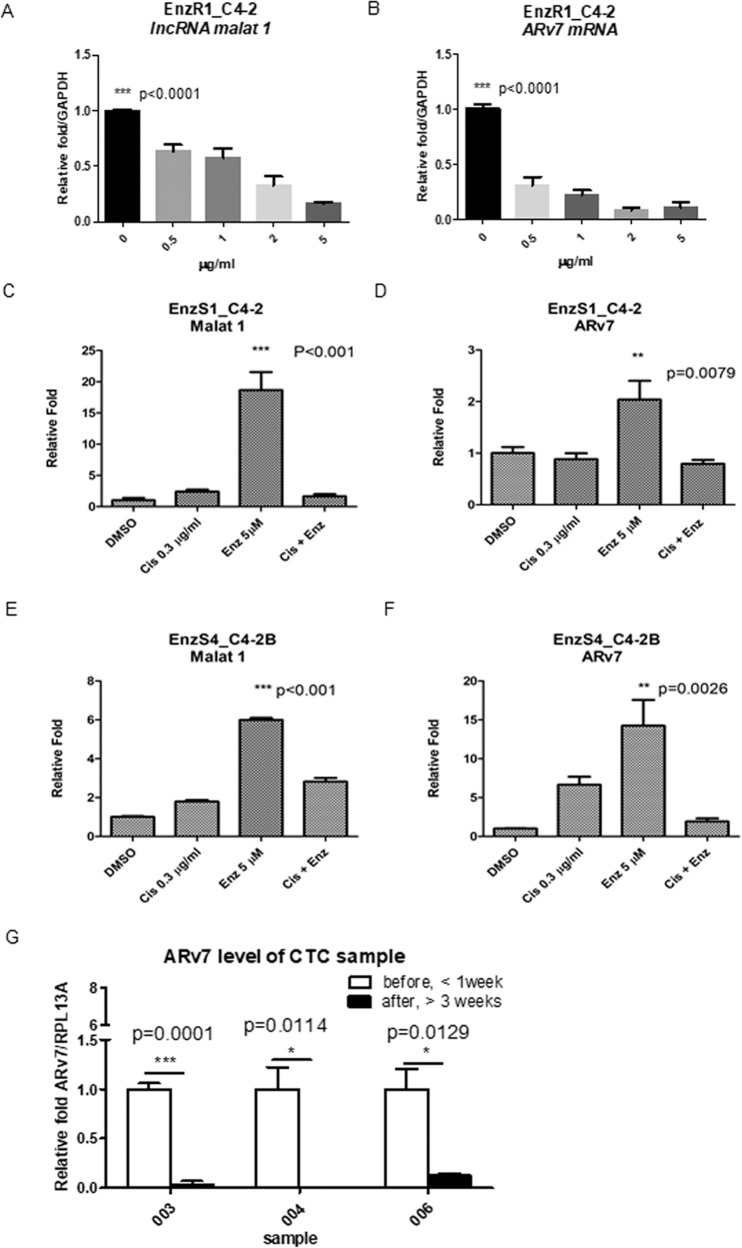
Cisplatin suppress Malat1/ARv7 pathway. EnzR1_C4-2 cells were treated with different Cis concentrations (0–5 μg/ml) for 24 h. **a**, **b** RNA level of *Malat1* (**a**) and ARv7 (**b**) In EnzS1_C4-2 cells, the RNAs expression level of Malat1 (**c**) and ARv7 (**d**) after treated with (DMSO, Cis 0.3 μg/ml, 5 μM Enz, Cis 0.3 μg/ml + 5 μM Enz) for 2 weeks. In EnzS4_C4-2B cells the RNAs expression level of Malat1 (**e**) and ARv7 (**f**) after treated with (DMSO, Cis 0.3 μg/ml, 5 μM Enz, Cis 0.3 μg/ml + 5 μM Enz) for 2 weeks. **g** Quantified PCR results of ARv7 at CRPC patients after Cis treatment. All Quantified PCRs were performed in triplicate. A-F multiple comparison was analyzed by One-way ANOVA. QPCR results presented as Mean ± SEM *P < 0.05, **P < 0.01, ***P < 0.001.

Since our recent studies indicated that Enz might function through inducing expression of the lncRNA-Malat1 and its associated SF2 RNA splicing protein (named as Malat1/SF2 RNA splicing complex) to increase ARv7 biosynthesis/expression^22^, we were interested to see if Cis may also function through altering this Malat1/SF2 RNA splicing complex to decrease the ARv7 mRNA biosynthesis/expression. As expected, the results revealed in EnzR1_C4-2 cells, treating with Cis led to decrease the mRNA expression of lncRNA-Malat1 and ARv7 in a dose-dependent manner (Fig. 5a, b). In contrast, we found Cis had little effect on the lncRNA-RP11-473I1.9 (Supplementary Fig. S1).

Furthermore, we found that treating EnzS1_C4-2 cells with 5 μM Enz alone for 2 weeks led to increase the expression of Malat1 and ARv7 (Fig. 5c, d, respectively), yet 10 μM Enz + 0.3 μg/ml Cis inhibited the increased expression of lncRNA-Malat1 and ARv7 (Fig. 5c, d, respectively). Similar results were also obtained when we replaced the EnzR1_C4-2 cells with the EnzS4_C4-2B (Fig. 5e, f) cells.

Together, the results shown in Fig. 5a–f demonstrate that Cis can also function through altering the Malat1/SF2 RNA splicing complex to decrease the ARv7 mRNA biosynthesis/expression.

In addition to functioning via altering the AR-ubiquitination to decrease ARv7/AR-F876L protein expression (See Fig. 2c) and modulating the Malat1/SF2 RNA splicing complex to decrease AR/ARv7 mRNA biosynthesis/expression (Fig. 5c, e), we were interested to see if Cis can also function via transcriptional modulation to decrease AR/ARv7 expression. We thus constructed the 3.6 kb *AR* promoter into pGL3 luciferase vector (p*AR*-luc) and transfected p*AR*-luc into EnzR3_CWR22Rv1 cells for luciferase assay. The results revealed that 1 μg/ml Cis could also decrease AR mRNA expression at the transcriptional level via altering AR promoter activity (Fig. S2).

Together, the results shown in Figs. 4 and 5 suggest that Cis may function via multiple mechanisms to degrade AR/ARv7 expression.

### Human clinical sample survey to demonstrate that Cis can degrade the fAR and ARv7

To prove that results described above, which were generated in the in vitro cell lines is recapitulated in human clinical samples, we also performed a small human clinical sample survey. We collected patients’ blood before and after Cis treatment, and isolated circulating tumor cells (CTCs) for mRNA purification. The quantitative PCR results revealed that Cis treatment in 3 out of 6 samples resulted in decreased ARv7 expression (Fig. 5g and Supplementary Fig. S3).

### Preclinical study using in vivo mouse model to prove Cis+ Enz can further suppress EnzR CRPC cells

**Fig. 6:**
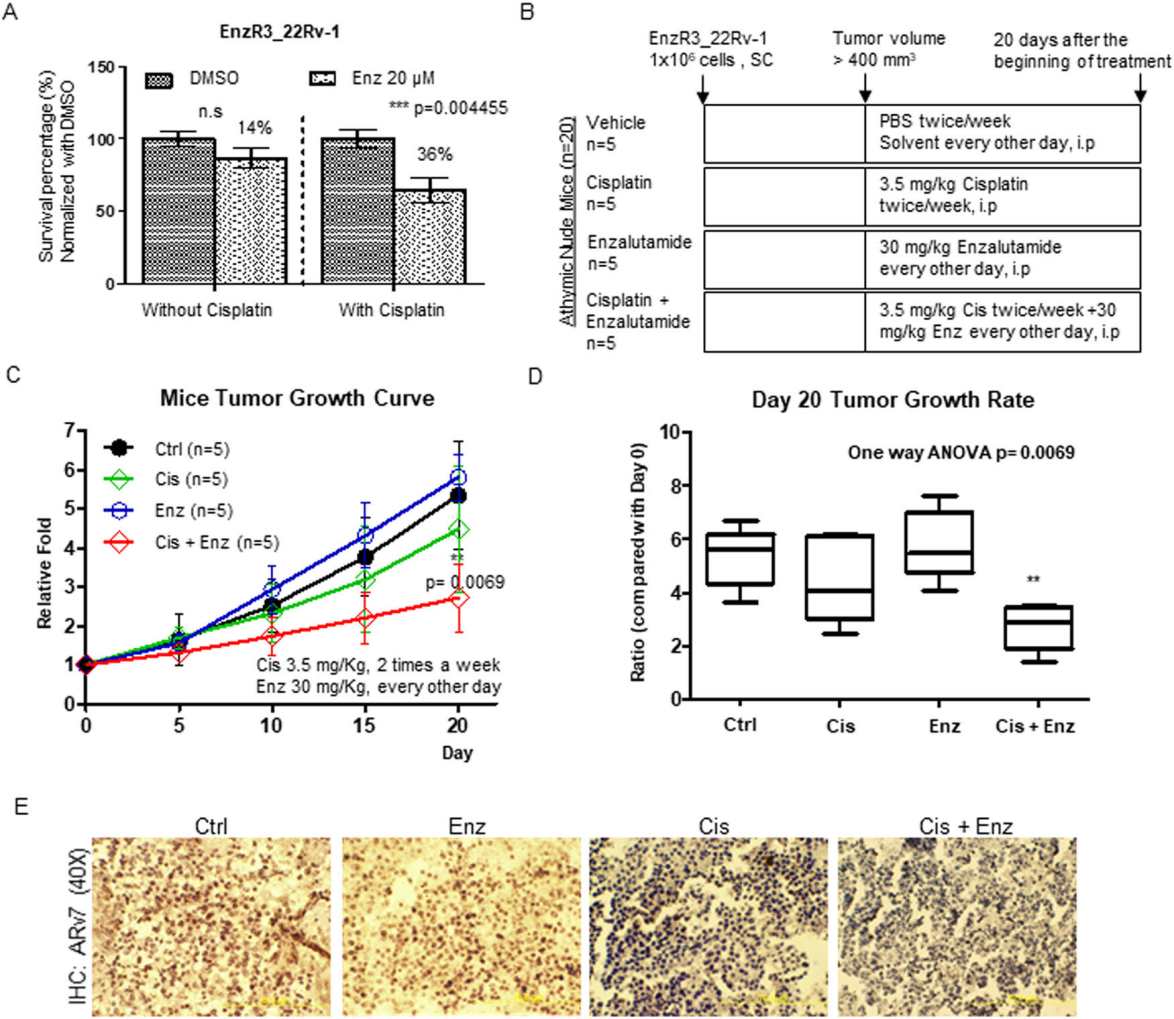
Cisplatin restores Enzalutamide sensitivity and inhibits the growth of EnzR tumors in pre-clinical mouse PCa model. **a** Survival rate of EnzR3_22Rv-1 cells with (w) or without (w/o) 1 μg/ml Cis treatment for 24 h and 20 μM Enz treatment for 6 days. **b** Schematic for athymic mice xenograft model treatment. **c** Tumor growth curve after 20 days in mice treated with DMSO (CTRL), 3.5 mg/kg Cis, 30 mg/kg Enz, or 3.5 mg/kg Cis + 30 mg/kg Enz (each group *n* = 5). **d** Box plot for day 20 tumor growth ratio. Comparison between multiple groups using One-way ANOVA. **e** Immunohistology staining result of ARv7 in different groups. Data presented as Mean ± S.D. *P < 0.05, **P < 0.01, ***P < 0.001.

Finally, to study the in vitro results in an in vivo model, we first confirmed most in vitro conclusions in the EnzR3_CWR22Rv-1 (EnzR_22Rv-1) cells that have better tumor growth rate in mice (Fig. 6a). Mice were subcutaneously implanted with EnzR3_CWR22Rv-1 cells. After PCa grew to ≥400 mm^3^ the mice were then i.p. injected with Enz (30 mM/kg/every other day) and with or without i.p. injections of Cis (3.5 mg/kg, 2 times a week) for 20 days^23^ and tumors measured weekly by calipers.

The results revealed that treating with Cis (sublethal dose, IC50 in Supplementary Fig. S4) could restore Enz sensitivity (from 14 to 36%) in the EnzR3_CWR22Rv1 cells (Fig. 6a). Based on our therapeutic procedure (Fig. 6b), the tumor growth rate showed the EnzR3_CW22Rv1 xenograft tumor growth could be significantly suppressed by Cis + Enz as compared with control, Enz, and Cis single treatment group (Fig. 6c, d), without any obvious unwanted effects (mice body weight Supplementary Fig. S5, and full size mice photo Supplementary Figs. S6–S7), and ARv7 IHC stainings are lower in Cis and Cis+Enz groups (Fig. 6e).

**Fig. 7:**
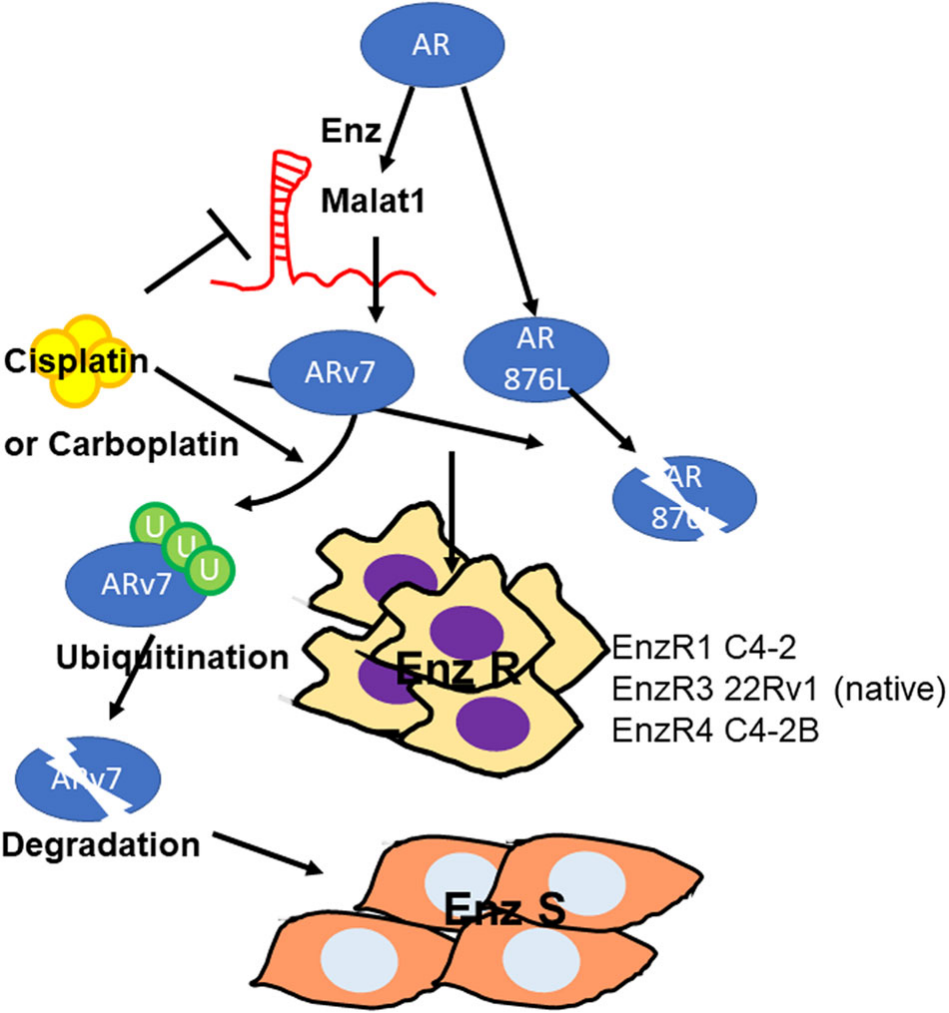
Schematic of ARv7/AR-F876L changes in EnzR cells under Cisplatin and Carboplatin treatment.

Together, the results from these preclinical studies using in vitro cell lines and in vivo mouse model (Fig. 6a–e) suggest that Cis can increase the Enz sensitivity to further suppress the EnzR cell growth (Fig. 7).

## Discussion

Most CRPC patients who receive Enz may develop the Enz-resistance, which might involve multiple mechanisms. For example, Enz or its 2^nd^ generation ARN509 might induce an AR point mutation at AR876 (a missense mutation of phenylalanine 876 to leucine in the Ligand-Binding-Domain (LBD) of AR, named AR-F876L)^24,25^ that is no longer sensitive to Enz treatment^24,25^. Early clinical data revealed that 3 of 29 CRPC patients receiving ARN509 treatment had the AR-F876L mutant^24–26^. Balbas et al.^26^ further confirmed these findings by showing that AR-F876L could convert Enz into an AR agonist and reverse the growth inhibition of Enz treatment.

Enz might also increase the glucocorticoid receptor (GR) signals^12^ in a subset of PCa cells due to relief of AR-mediated feedback repression of GR expression^27^. GR and AR are closely related members of the nuclear receptor superfamily with a similar DNA-binding-domain, and GR could replace part of AR roles during development of Enz-resistance^28^. Importantly, Arora et al.^12^ found the GR agonist dexamethasone was sufficient to confer Enz-resistance, whereas a GR antagonist could partially restore sensitivity.

However, the emergence of the AR splicing variant ARv7 may represent the key factor for the development of Enz-resistance as recent clinical studies from CRPC patients demonstrated that 39% of metastatic CRPC patients treated with Enz had detectable ARv7 in their circulating tumor cells^8^, and these ARv7 positive patients had lower PSA response rates than ARv7 negative patients with shorter PSA progression–free survival (median, 1.4 months vs. 6.0 months), suggesting CRPC patients with ARv7 might have poor response to Enz treatment, and Enz treatment might enhance ARv7 expression^8^. ARv7 is constitutively active and reported to regulate a transcriptional program that is similar, but not identical, to that of AR in CRPC^29–32^, and treating with Enz could increase the expression of constitutively active ARv7^9^ that might transactivate AR target genes to promote CRPC progression in an androgen-independent manner^33^.

At least 2 newly developed compounds have been demonstrated to be able to target the ARv7. The first one is the AR degradation enhancer ASC-J9^®^, that was shown to selectively degrade AR protein in some, but not all cell types^34^, with fewer adverse effects in all in vivo mice studies^31,33–40^. Importantly, ASC-J9^®^, but not the anti-androgens Enz or Casodex, could degrade both full-length AR and the AR variant ARv7 or AR mutants including AR-F876L^31,33^.

Niclosamide, an anti-helminthic compound, is the 2^nd^ compound identified as an ARv7 inhibitor to suppress the PCa progression^41^. Liu et al. found niclosamide could suppress ARv7 protein expression via a proteasome-dependent pathway to suppress the PCa cell growth in vitro and in vivo^41^.

The microRNA (miR), miR-124, has recently been identified as a tumor suppressor to suppress the PCa progression^42^, and miR-124 could also suppress ARv7 along with EZH2 and Src signals^42^.

While all 3 compounds mentioned above may have the capacity to target the ARv7 in various in vitro PCa cell lines and in vivo models, none of these compounds are ready to be used in CRPC patients that already developed Enz-resistance. In contrast, Cis or Carboplatin have been widely used as chemotherapy in various tumors, including PCa^43,44^, since its approval in 1978 by the U.S. Food and Drug Administration. Currently, medical oncologists apply Cis chemotherapy in common clinical practice to treat various tumors with either 70 mg/m^2^ every 3 weeks or 20 mg/m^2^ daily × 5 days every 3 weeks or 20 mg/m^2^ weekly (equal to 6.75 mg/kg mouse)^20,45–47^. Our effective Cis dose used in the in vivo model here is 3.5 mg/kg mouse that is less than the allowed (extrapolated to human) dose used to treat human tumor patients.

Interestingly, all previous studies for the Cis effect to suppress tumor progression focused on its capacity to cross-link to DNA that resulted in triggering apoptosis and interfering with the process of cell division^17^. Its linkage to alter the AR signals, especially the degradation of AR mutant ARv7, however, remains unclear. Our results showing Cis may function via degrading ARv7 to restore/increase Enz sensitivity to further suppress EnzR cell growth and to delay the Enz-induced Enz-resistance in CRPC may have clinical relevance to be immediately used to treat those CRPC patients who received Enz treatment and developed Enz-resistance.

The potential adverse effects of Cis treatment include myelosuppression, asthenia and gastrointestinal disorders, as well as long-term cardiac, renal and neurological consequences, which may result in its discontinuation and limit its therapeutic efficacy^48,49^. Here, we demonstrate there are therapeutic windows that can suppress lncRNA-Malat1 function and enhance ARv7 degradation before cell apoptosis, which means it is possibly able to increase Enz therapeutic efficacy via ARv7 degradation without carrying those unwanted adverse effects.

Alternatively, we may be able to use Carboplatin to replace Cis since Carboplatin shares similar anti-tumor effects with Cis, but has less adverse effects than Cis^50^. Importantly, Carboplatin like Cis, can also target ARv7 (see Fig. 1g) as well as to further suppress EnzR cell proliferation (see Fig. 2g–i).

In conclusion, our results from using multiple CRPC cell lines, a mouse model, and human CTC samples, not only reveal unrecognized mechanisms by which Cis can degrade ARv7/AR-F876L via suppressing the lncRNA-Malat1/ARv7 and boost ubiquitination signals (Fig. 7), they may also provide a novel and ready therapy to suppress the EnzR CRPC cells to further extend CRPC patients survival.

## Supplementary information

Supplementary legends

Fig S1

Fig S2

Fig S3

Fig S4

Fig S5

Fig S6

Fig S7
