# Supplementary material for: Preclinical studies using cisplatin/carboplatin to restore the Enzalutamide sensitivity via degrading the androgen receptor splicing variant 7 (ARv7) to further suppress Enzalutamide resistant prostate cancer
Source: Cell Death Dis. 2020 Nov 2;11(11):942. doi: 10.1038/s41419-020-02970-4 (PMC7606511; doi:10.1038/s41419-020-02970-4)
Supplement: Supplementary file 1 — Supplementary legends [file 41419_2020_2970_MOESM1_ESM.docx]

**Supplementary legends**

**Figure S1.** The *IncRNA RP-11-473/1.9* (as negative control) expression levels after increasing doses of Cisplatin treatment.

**Figure S2.** AR promoter activity decreased after Cisplatin treatment.

**Figure S3** ARv7 QPCR results in CTC samples (did not fit hypothesis).

**Figure S4** The IC50 of EnzR3_22Rv-1 under Cisplatin treatment.

**Figure S5** Mice body weight ratio after/before Cis treatment.

**Figure S6** Full size of mice data (Ctrl and Enzalutamide)

**Figure S7** Full size of mice data (Cis and Cisplatin + Enzalutamide)
