# Supplementary figures and images for: Preclinical studies using cisplatin/carboplatin to restore the Enzalutamide sensitivity via degrading the androgen receptor splicing variant 7 (ARv7) to further suppress Enzalutamide resistant prostate cancer
Source: Cell Death Dis. 2020 Nov 2;11(11):942. doi: 10.1038/s41419-020-02970-4 (PMC7606511; doi:10.1038/s41419-020-02970-4)

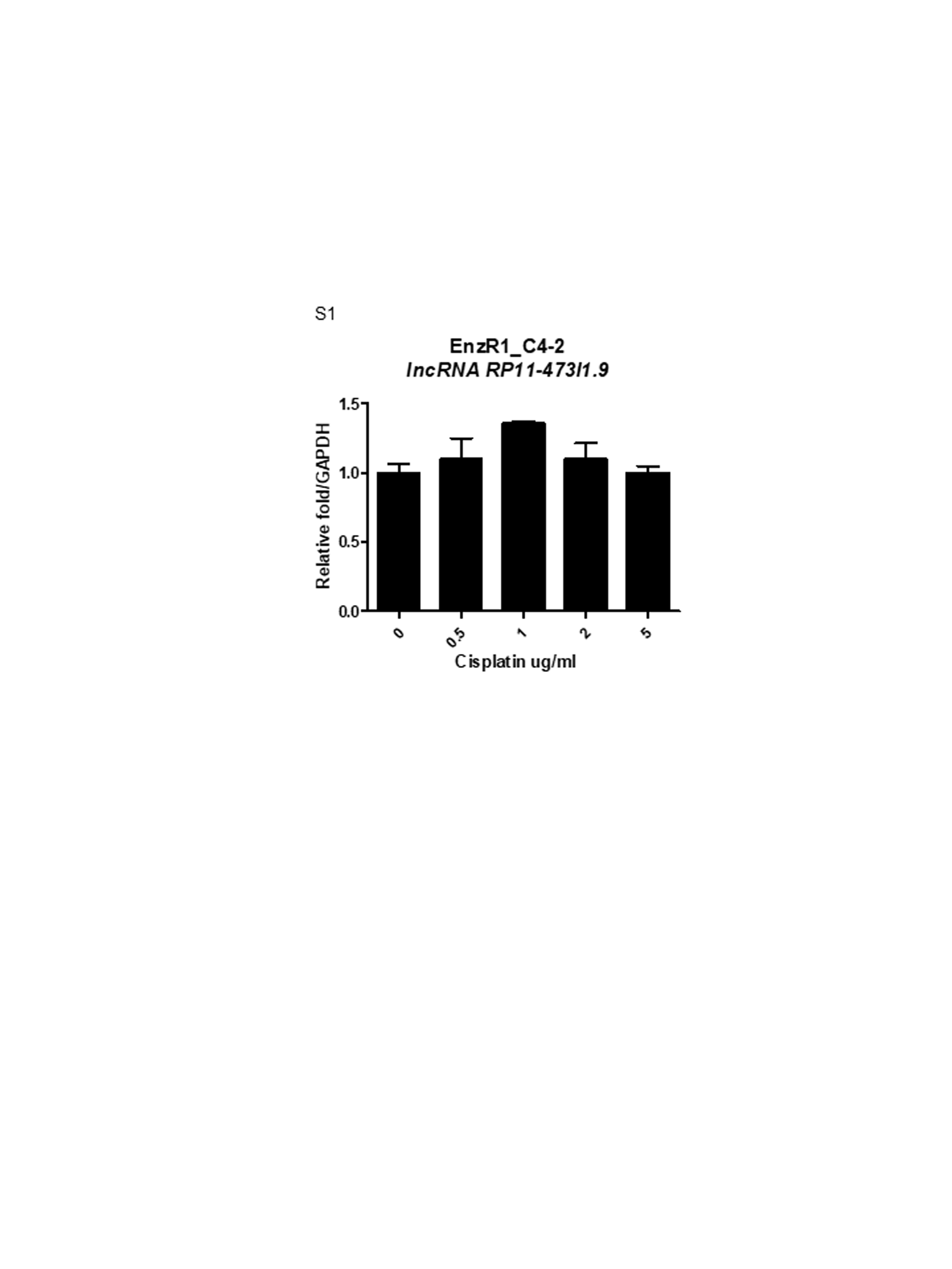

Supplement: Supplementary file 2 — Fig S1 [file 41419_2020_2970_MOESM2_ESM.tif]

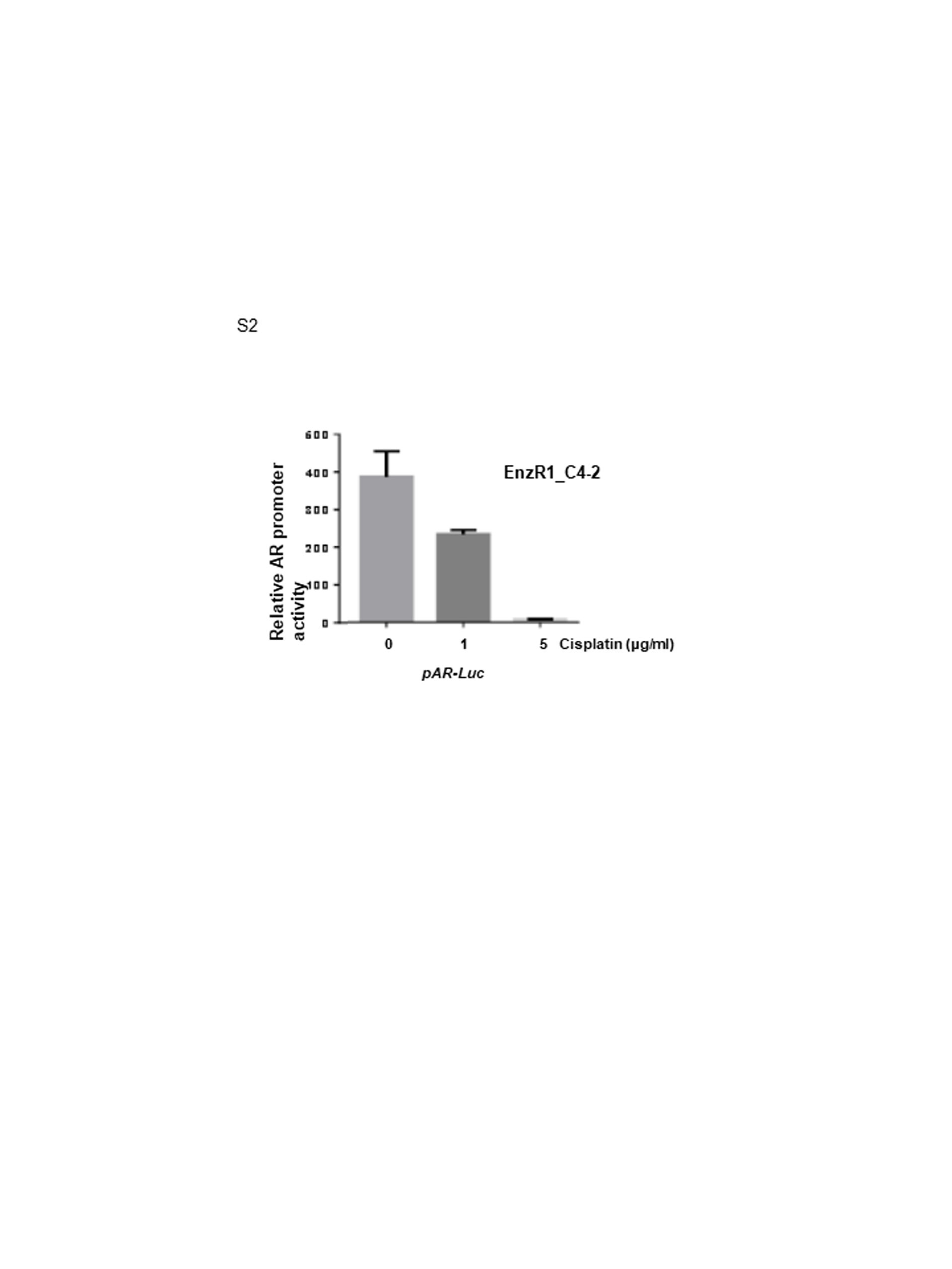

Supplement: Supplementary file 3 — Fig S2 [file 41419_2020_2970_MOESM3_ESM.tif]

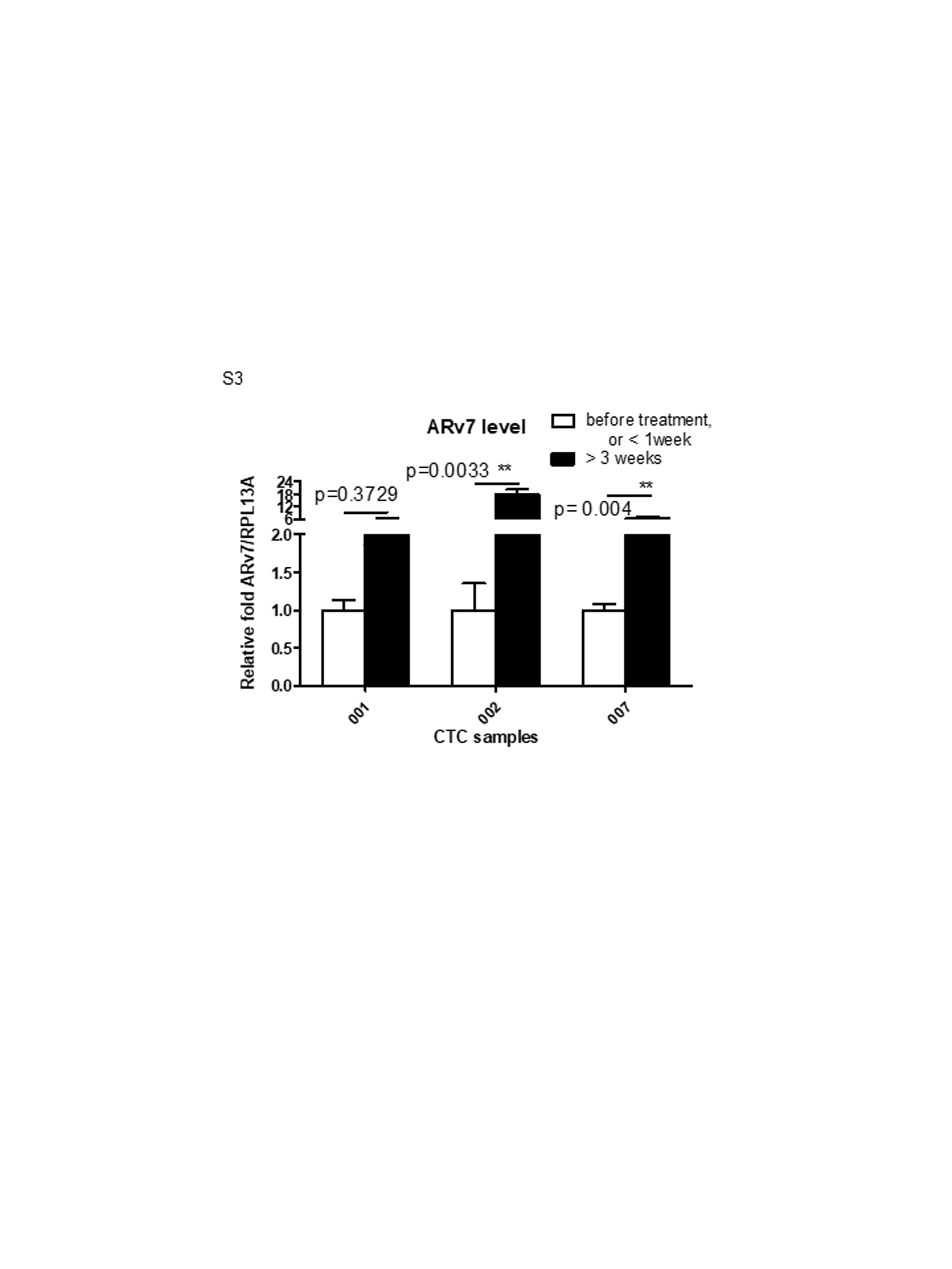

Supplement: Supplementary file 4 — Fig S3 [file 41419_2020_2970_MOESM4_ESM.tif]

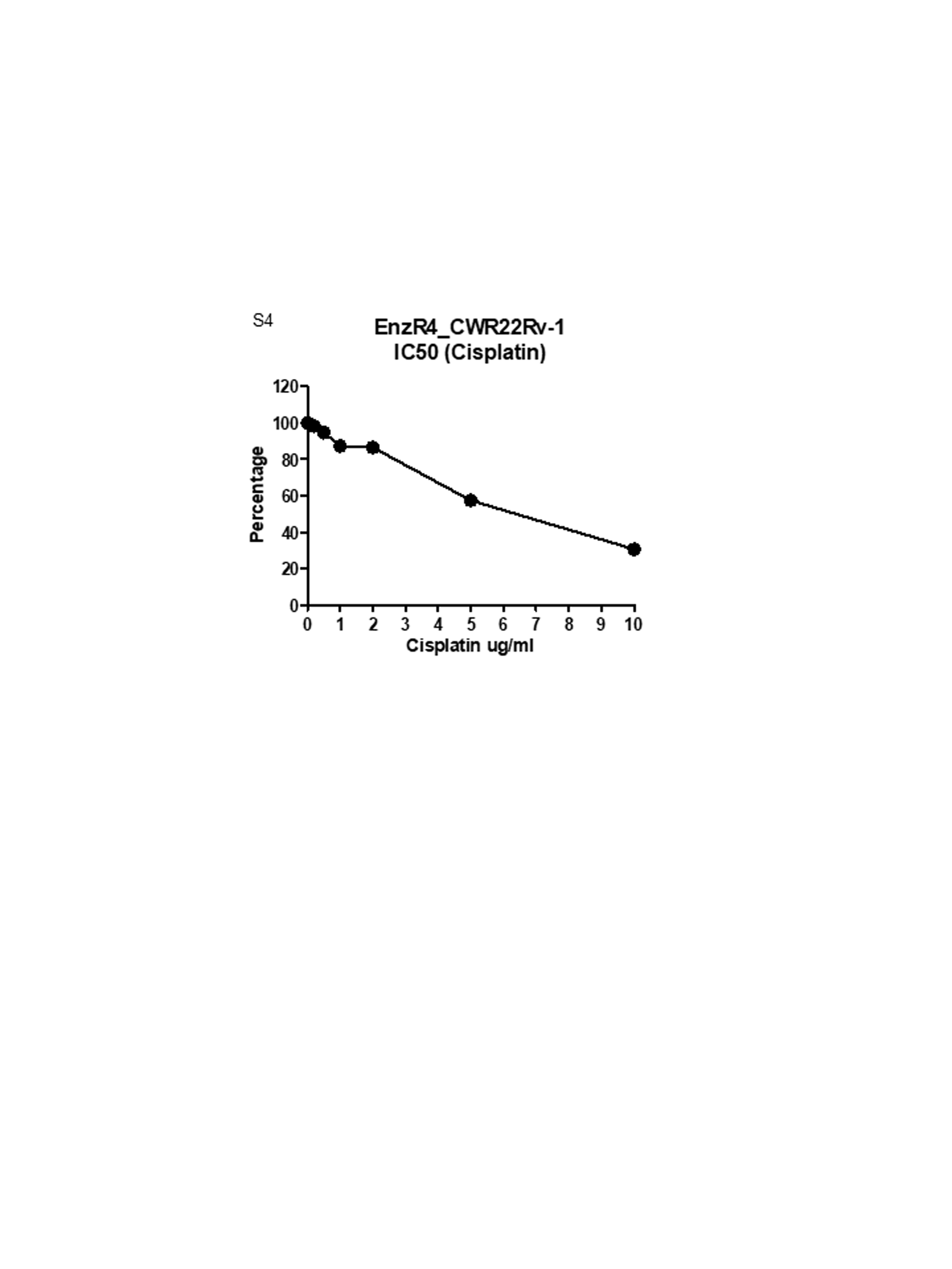

Supplement: Supplementary file 5 — Fig S4 [file 41419_2020_2970_MOESM5_ESM.tif]

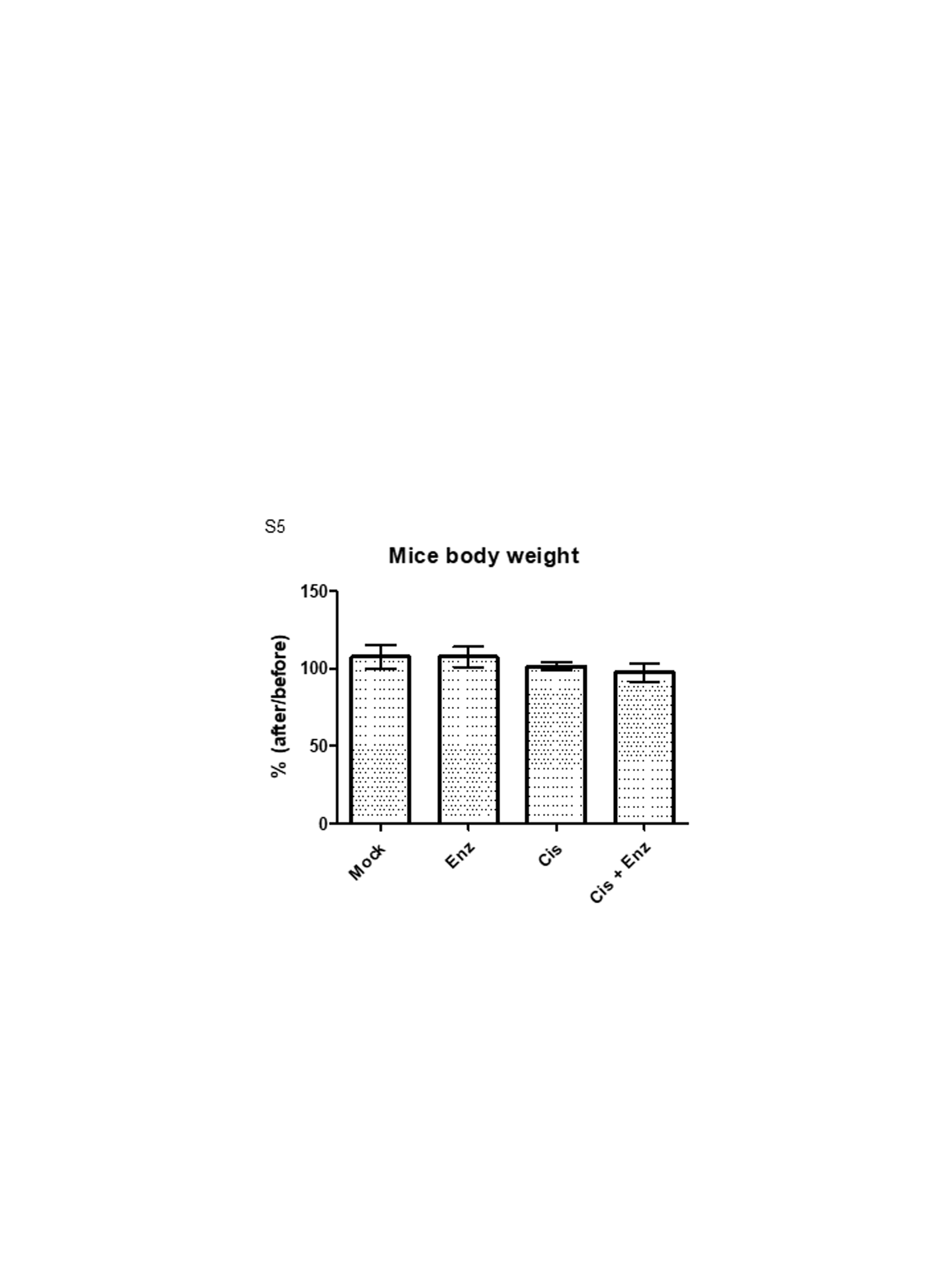

Supplement: Supplementary file 6 — Fig S5 [file 41419_2020_2970_MOESM6_ESM.tif]

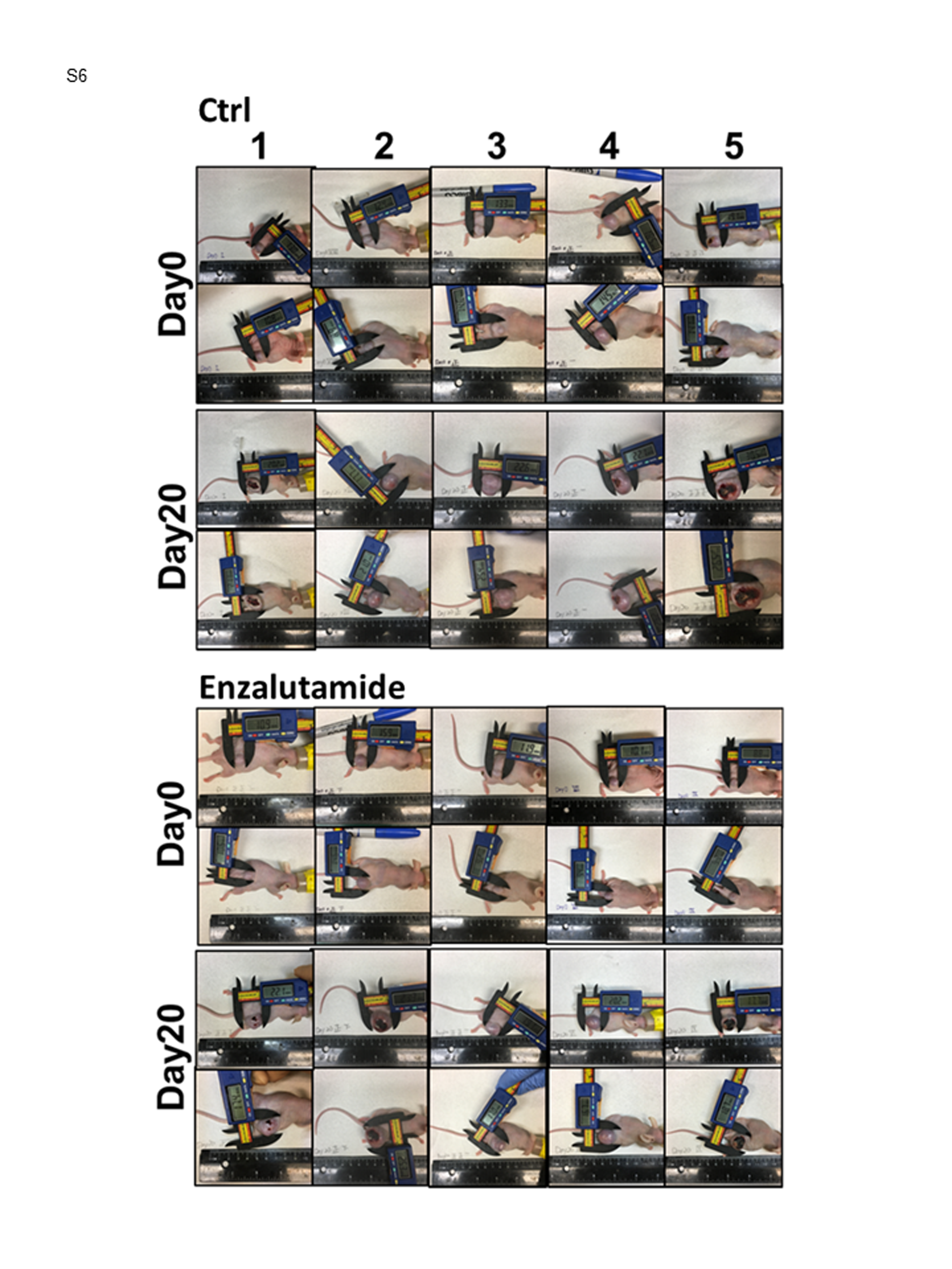

Supplement: Supplementary file 7 — Fig S6 [file 41419_2020_2970_MOESM7_ESM.tif]

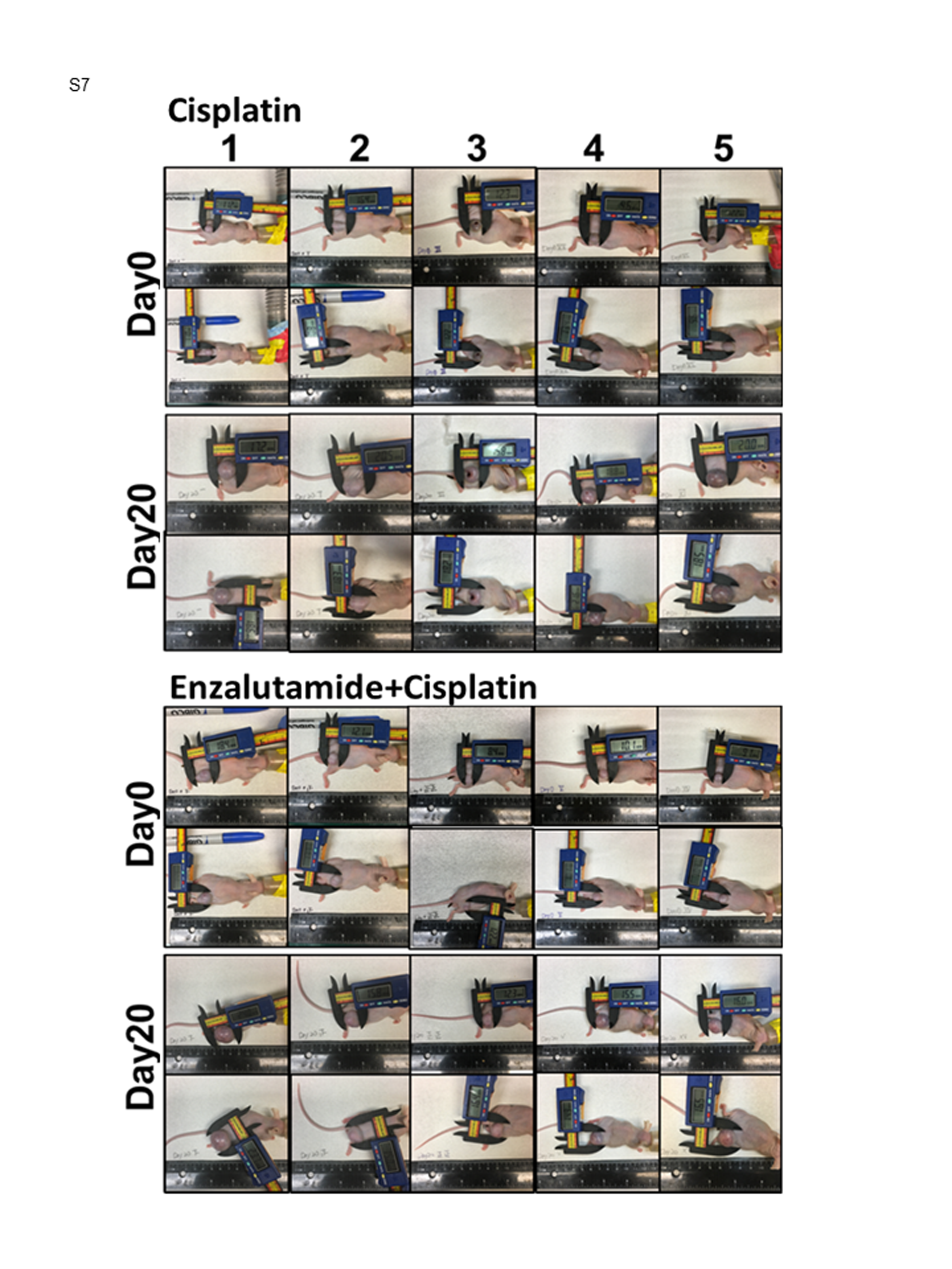

Supplement: Supplementary file 8 — Fig S7 [file 41419_2020_2970_MOESM8_ESM.tif]
